# The four-domain structure model of a depression scale for medical students: A cross-sectional study in Haiphong, Vietnam

**DOI:** 10.1371/journal.pone.0194550

**Published:** 2018-03-22

**Authors:** Thao Thi Thu Nguyen, Ngoc Thi Minh Nguyen, Manh Van Pham, Han Van Pham, Hiroyuki Nakamura

**Affiliations:** 1 Department of Environmental and Preventive Medicine, Graduate School of Medical Sciences, Kanazawa University, Kanazawa City, Ishikawa Prefecture, Japan; 2 Department of Environmental Health, Public Health Faculty, Haiphong University of Medicine and Pharmacy, Haiphong City, Vietnam; 3 Department of Psychiatry, Haiphong University of Medicine and Pharmacy, Haiphong City, Vietnam; Universidade de Mogi das Cruzes, BRAZIL

## Abstract

Depression is a common mental health problem with a higher prevalence in medical students than in the general population. This study aims to investigate the association between depressive symptoms, particularly those in each domain of the *Center for Epidemiological Studies Depression (CES-D)* Scale, and related factors. A cross-sectional study was conducted with a random sample of 1319 medical students at Haiphong University of Medicine and Pharmacy in 2016. The CES-D scale and a self-reported questionnaire were used to identify the prevalence of depressive symptoms and related risk factors. Univariate and multivariate logistic regression were performed to assess the risk factors associated with depressive symptoms and the score for each structure factor. Depressive symptoms were observed in 514 (39%) students, including more males than females (44.2% vs 36.9%, p = 0.015). Students whose mothers’ highest education level was primary school had a higher prevalence of depressive symptoms than students whose mothers had higher education levels (p = 0.038). There was a significant relationship between depressive symptoms and stressful life events, especially a decline in personal health. A higher correlation was found between the somatic complaints and depressive affect domains. The impacts of risk factors differed for each domain of the depression scale. Only the factor of achieving excellence showed no statistically significant associations with depressive symptoms and the scores on the four domains considered in this study. The high prevalence of depressive symptoms among medical students with risk factors and the impact of these risk factors on each domain of depression scale need further clarification to alleviate depression in students during their medical training.

## Introduction

Depression, a well-known mental health concern, is ranked by the World Health Organization (WHO) as the single largest contributor to global disability and a major contributor to suicide deaths[1]. Globally, the total number of people with depression was estimated to exceed 300 million in 2015 and to have increased by 18.4% in the 10 years since 2005[2]. Depression presents as persistent sadness, a loss of interest and energy, an inability to carry out daily activities, a change in appetite or sleeping time, poor concentration, hopelessness and thoughts of self-harm or suicide[3]. It is a consequence of complex interactions among many factors. Among adolescents, a combination of biological, psychological and environmental factors has been documented to increase the risk of depression[4]. In addition, sociodemographic characteristics, such as age, income, parenting style, living conditions and parent education, have also been associated with depression[5–7].

Medical school is a time of significant psychological distress for physicians in training. The school applies an extensive selection process to identify intelligent and altruistic individuals with a strong commitment to medical education and then spends years trying to prepare those individuals to achieve their goals. Medical students face numerous common daily life stressors in addition to stress specifically arising from longstanding academic demands, such as high workloads and sleep deprivation, which create a work environment that can lead to the development of mental health problems[8]. Previous studies have suggested a high prevalence of depression and anxiety among medical students[9]. However, the prevalence varies across studies. Research on medical students at the University of Copenhagen showed that nearly one-third of the participants reported feeling depressed[10], a prevalence similar to those reported by studies in Nepal and Brazil[7, 11]. A systematic review examining depression, anxiety and distress in medical students outside North America reported a variety of depression rates ranging from 6.0 to 66.6%[12]. The overall pooled crude prevalence of depressive symptoms was 27.2%[13]. Greater severity of depression was observed in medical students who were female, were intrinsically religious, lived in a hostel or a rented house, experienced major life events and were at the clinical grade level [7, 11, 14]. However, such associations were not seen in other studies[15].

To prevent depressive symptoms among medical students, factors associated with depression in medical training and in the students’ daily activities, should be identified and approached appropriately. There are 9 large medical universities in Vietnam (excluding newly established faculties of medicine at some universities), and the general curriculum at each university follows a national framework but tends to vary from university to university. Medical training lasts either 4 or 6 years, with competitive graduates attending residency programs following graduation[16]. As a general schedule, after studying at the university for the first 2 or 3 years (mainly focusing on basic science subjects), students spend their final 2 or 3 years studying and practicing in hospitals. Students must take examinations to graduate from each university, but they are not required to take a national licensing exam. In Vietnam, the number of doctors has increased over the years, reaching 11.9 doctors per 100,000 people in 2015[17]. The health care workforce continues to grow. Shortages remain in some fields, such as pathology, but there are no longer problems with severe shortages, except in the field of mental health[18]. Therefore, little is known about the trajectory of depression, especially among medical students. Additionally, Vietnamese studies of depression not only in students but also in the general population have encountered many limitations. Thus, the statistics for depression and other mental health problems are not well known. The *Center for Epidemiological Studies Depression* (CES-D), a 20-item inventory with four domains, which was developed by the National Institute of Mental Health Center for Epidemiology Studies, has been widely used to measure depressive symptoms[19]. The aim of this study was to investigate the prevalence and risk factors associated with depressive symptoms among medical students at Haiphong University of Medicine and Pharmacy (HPUMP), Vietnam, throughout their medical education, particularly to determine whether there were changes in each domain that the depression scale assesses.

## Materials and methods

### Ethics statement

We followed all national standards and laws for research in humans and obtained the necessary permission to conduct this study. The research protocol was approved by the HPUMP Review Board in Bio-Medical Research, the ethics committee of HPUMP (number 06/HPUMPRB, approved in December 2015). Written informed consent was obtained from all study participants.

### Participants and procedures

This cross-sectional observational study was performed at HPUMP, one of five medical universities in Northern Vietnam (the others are Hanoi Medical University, Thai Nguyen Medical University, Thai Binh Medical University, and Vietnam Military Medical Academy). For the 2015–2016 school year, HPUMP had 5822 undergraduate students, including 4093 regular students, 881 students attending crash courses (course for students who have already had jobs related to the medical field) and 847 in-service students [20]. The present study included only undergraduate regular students. All the classes in each year (from the 1^st^ year to the 6^th^ year) were listed: the 1^st^ year comprised 14 classes; the 2^nd^, 3^rd^, and 4^th^ years comprised 13 classes each; and the 5^th^ and 6^th^ years included 11 classes each. The study requested a schedule for each class from the Student Office. The staff at the Student Office helped to pick up the class for investigating. Depending on the schedule (when all the students of one class could gather for a lecture at the university), we visited classes and recruited students after their classes finished. Students were recruited from four randomly selected classes in each grade. A total of 1359 students in different semesters and majors who agreed to participate in this study were chosen and given questionnaires (response rate was 100%). After the data were cleaned, 40 students (3%) were excluded because of missing data. Finally, the data of 1319 undergraduate students were used for the analysis.

The self-reported questionnaire was given to each student to complete after classes. The questionnaire comprised three parts: 1) Sociodemographic data (gender and age), specialization (general medicine or other: preventive medicine, medical technicians, nursing, odonto-stomatology, pharmacy), grades (pre-clinical and clinical grades), hometown area (suburb or urban), living condition (dormitory, rental house, or with family), marital status (single or other: engaged, married, or divorced), parents’ highest educational background, and parents’ marital status; 2) Depression-related factors (including stressful life events items such as conflict with parents, financial problems, death of a close family member, lower grades than expected [21] and others: health problems, whether they would chose a medical career again, conflict with teachers, etc.); and 3) CES-D scale.

### Measures

The CES-D scale consists of 20 calibrated items designed to measure depressive symptoms in the general population[19]. The CES-D has a four-domain structure comprising depressed affect (7 items), somatic complaints (7 items), positive affect (4 items) and interpersonal activity (2 items). Specific depression symptom factors have implications for both assessment and treatment. It is often suggested that patients who have different symptom profiles are likely to have different prognoses and may require different treatment[22]. Each item is scored ranging from zero to three to indicate how often the symptom occurred during the past week: rarely or none of the time (0), some or a little of the time (1), occasionally or a moderate amount of the time (2) and most of the time (3). Total scores on the CES-D scale range from 0 to 60 points, with a higher score indicating a higher level of depression. The standard cut-off score of 16 was used to detect cases with depressive symptoms. A Vietnamese version of the CES-D that was validated in Vietnamese adolescents with a high internal consistency (Cronbach’s alpha of 0.84)[23] was used to measure depressive symptoms.

### Statistical analyses

The collected data were recorded in Excel for Windows, and statistical analyses were performed using the software package SPSS version 19.0 (SPSS, Inc.). The chi-square test was used to assess the significance of differences in the distribution of selected sociodemographic characteristics. Logistic regression models were employed to assess the factors associated with dependent variables (depression and each domain). All sociodemographic variables that had p<0.05 in the univariate analysis were used as confounding factors in the multivariate analysis (S1 Table). A value of p<0.05 and a 95% confidence interval were used for all analyses.

## Results

### Sociodemographic characteristic

A total of 1319 students participated in the study, and 514 (39%) reported depression. The prevalence of depressive symptoms in the male students was 44.2% (164/371), which was significantly higher than the prevalence in females (36.9%, 350/948). Among the students, 61.8% were younger 20 years of age, and only 4.6% were older than 25 years of age. More than 90% of the students were Kinh people and had no religious affiliation. Regarding location of residence, 81.4% of the students were from the suburbs, and 75% lived in rental houses. The number of students in the general medicine course was 511 (38.1%), which was a smaller proportion than that that of students in specializations (808 participants; 61.3%). There were 54.1% students in the pre-clinical grade level and 45.9% practicing in the hospital. Only 3.9% of the students were married or living with partners. Most of the students had fathers and mothers with an educational background of secondary school. Of the students whose mothers’ highest education level was primary school, 52.6% had depressive symptoms, a significantly higher proportion than other groups. Sample characteristics are shown in Table 1.

**Table 1:** Participant characteristics.

Characteristics		Total (n = 1319)	Depressive symptoms status (n,%)		p*
			Non-depressive symptoms (805, 61%)	Depressive symptoms (514, 39%)	
Gender	Male	371(28.1%)	207(55.8%)	164(44.2%)	0.015
	Female	948(71.9%)	598(63.1%)	350(36.9%)	
Age (years)	≤ 20	815(61.8%)	499 (61.2%)	316 (38.8%)	0.845
	21–24	443(33.6%)	267 (60.3%)	176 (39.7%)	
	≥25	61(4.6%)	39 (63.9%)	22 (36.1%)	
Ethnicity	Kinh	1244(94.3%)	764(61.4%)	480(38.6%)	0.245
	Non-Kinh	75(5.7%)	41(54.7%)	34(45.3%)	
Religion	None	1210(91.7%)	746(61.7%)	464(38.3%)	0.123
	Religious	109(8.3%)	59(54.1%)	50(45.9%)	
Area	Urban	245(18.6%)	149(60.8%)	96(39.2%)	0.939
	Suburban	1074(81.4%)	656(61.1%)	418(38.9%)	
Living condition	Dormitory	118(8.9%)	70(59.3%)	48(40.7%)	0.176
	Rental house	989(75.0%)	617(62.4%)	372(37.6%)	
	With family	212(16.1%)	118(55.7%)	94(44.3%)	
Marriage status	Single	1267(96.1%)	777(61.3%)	490(38.7%)	0.278
	Other	52(3.9%)	28(53.8%)	24(46.2%)	
Specialization	General medicine	511(38.1%)	322(63.0%)	189(37.0%)	0.240
	Other	808(61.3%)	483(59.8%)	325(40.2%)	
Grade level	Pre-clinical	713(54.1%)	427(59.1%)	286(40.9%)	0.356
	Clinical	606(45.9%)	378(62.4%)	228(37.6%)	
Fathers’ educational background	≤Primary school	80(6.1%)	48(60.0%)	32(40.0%)	0.149
	Secondary school	443(33.6%)	252(56.9%)	191(43.1%)	
	High school	447(33.9%)	281(62.9%)	166(37.1%)	
	>High school	349(26.5%)	224(64.2%)	125(35.8%)	
Mothers’ educational background	≤Primary school	78(5.9%)	37(47.4%)	41(52.6%)	0.038
	Secondary school	481(36.5%)	286(59.5%)	195(40.5%)	
	High school	447(33.9%)	282(63.1%)	165(36.9%)	
	>High school	313(23.9%)	200(63.9%)	113(36.1%)	
Parents’ marital status	Living together	1223(92.7%)	745(60.9%)	748(39.1%)	0.759
	Other	96(7.3%)	60(62.5%)	36(37.5%)	

*Chi-square test

### Factors associated with depressive symptoms

Univariate and multivariate logistic regression analyses were performed to study the association between depressive symptoms and related factors. The risk of depressive symptoms in female students was 0.739 times lower (95%CI: 0.579–0.943) than in male students. Students whose mothers’ highest educational background was primary school had a significantly higher risk of experiencing depressive symptoms compared with those whose mothers had a higher educational level (S1 Table). Students were likely to show increased depressive symptoms if they felt an increase in pressure to study (OR [95%CI]: 1.595 [1.241–2.049]). Those who received lower grades than expected, had been absent from many classes, felt that they would choose this career again, or had a conflict with teachers or a university policy offense also showed increased depressive symptoms (OR[95%CI] of 1.332 [1.049–1.690], 1.846 [1.332–2.559], 1.543 [1.048–2.272], 2.631 [1.604–4.314] and 2.371 [1.516–3.707], respectively). Students who experienced a decline in their personal health had a 2.683 greater chance of having depressive symptoms than those who had not. Students who reported conflicts with their parents, had experienced the death of a close family member, had difficulty participating in social activities or were uncomfortable with their living situation also showed significantly higher levels of depressive symptoms (OR [95%CI] of 1.942 [1.482–5.547], 1.821[1.299–2.552], 1.884[1.505–2.359] and 2.213 [1.740–2.814], respectively). The significant relationships were retained for the multivariate analysis. In contrast, there was no statistically significant association between depressive symptoms and the achievement of excellent study results (OR: 0.989; 95%CI: 0.664–1.475) or financial difficulty (OR: 1.186; 95%CI: 0.949–1.484). There were no significant changes in the statistics using multivariate logistic regression (Table 2).

**Table 2:** The associations of depressive symptoms with related factors.

	Univariate			Multivariate		
Factors	OR	95% C.I. for OR		OR	95% C.I. for OR	
		Lower	Upper		Lower	Upper
Increased pressure to study *	1.595	1.241	2.049	1.643	1.274	2.118
Lower grades than expected **	1.332	1.049	1.690	1.371	1.072	1.753
Achieving excellence in studies	0.989	0.664	1.475	0.975	0.652	1.457
Being absent from many classes *	1.846	1.332	2.559	1.828	1.313	2.545
Conflict with teachers *	2.631	1.604	4.314	2.650	1.602	4.383
Would choose medical career again **	1.543	1.048	2.272	1.576	1.063	2.337
University policy offence *	2.371	1.516	3.707	2.276	1.449	3.576
Experiencing a decline in personal health *	2.683	2.132	3.376	2.740	2.171	3.458
Financial difficulty	1.186	0.949	1.484	1.197	0.953	1.503
Conflict with parents *	1.942	1.482	2.547	1.987	1.511	2.614
Death of a close family member *	1.821	1.299	2.552	1.823	1.297	2.561
Difficulty participating in social activities*	1.884	1.505	2.359	1.956	1.556	2.459
Uncomfortable with living situation*	2.213	1.740	2.814	2.256	1.770	2.875

* p≤0.001

**p<0.05

Multivariate analysis adjusted for gender, age, ethnicity, religion, marital status, specialization. OR: Odds ratio, C.I.: Confidence interval

### Factors associated with each domain of the CES-D scale

All four domains had positive significant associations with one another, and the correlation between somatic complaints and depressive affect was the strongest (r^2^ = 0.734). These two domains also had a stronger correlation with the overall depression score than the other domains did (somatic complaints: r^2^ = 0.854, depressive affect: r^2^ = 0.897; Table 3).

**Table 3:** Pearson correlations for the four domains of the depression scale.

Domain (mean score ± SD)	Somatic complaints	Depressive affect	Positive affect	Interpersonal problems
Somatic complaints (4.80±3.67)	1			
Depressive affect (3.85±4.15)	0.734^**^	1		
Positive affect (5.15±3.34)	0.321^**^	0.392^**^	1	
Interpersonal problems (0.91±1.29)	0.483^**^	0.528^**^	0.239^**^	1
Depression score (14.71±9.84)	0.854^**^	0.897^**^	0.656^**^	0.615^**^

** Correlation is significant at the 0.01 level (2-tailed).

Being absent from many classes, having conflicts with teachers, experiencing a decline in personal health, having conflicts with parents, having difficulties participating in social activities and feeling uncomfortable with one’s living situation were factors that had positive significant associations with all four domains. Experiencing a decline in personal health had the strongest effect on the somatic complaints and depressive affect domains compared with other factors, with ORs of 2.345 and 2.110, respectively; the second strongest impact was that the effect of a university policy offense on these domains (OR of 2.108 and 2.086, respectively). Achieving excellence in study did not have a significant relationship with any of the four domains. Financial difficulty only significantly affected the somatic complaints domain (B = 0.734, p<0.001) and the depressive affect domain (B = 0.947, p<0.001). The positive affect domain was less affected by the factors than the other domains were; only six of the 13 factors had a significant association (Table 4).

**Table 4:** Association between depression risk factors and the four domains of the CES-D scale.

Factors	Somatic complaints			Depressive affect			Positive affect			Interpersonal problems		
	B	SE	p-value	B	SE	p-value	B	SE	p-value	B	SE	p-value
Increased pressure to study	0.877	0.220	<0.001	1.011	0.249	<0.001	0.259	0.203	0.202	0.230	0.078	0.003
Lower grades than expected	0.777	0.217	<0.001	1.067	0.245	<0.001	0.058	0.200	0.770	0.112	0.077	0.147
Achieving excellence in studies	-0.007	0.361	0.984	-0.223	0.409	0.585	0.026	0.331	0.937	0.215	0.128	0.093
Being absent from many classes	0.696	0.303	0.022	1.291	0.342	<0.001	0.917	0.277	0.001	0.393	0.107	<0.001
Conflict with teachers	1.013	0.451	0.025	2.073	0.508	<0.001	1.275	0.412	0.002	0.851	0.158	<0.001
Would choose medical career again	0.581	0.361	0.108	0.447	0.409	0.275	0.010	0.331	0.975	0.519	0.127	<0.001
University policy offence	2.108	0.406	<0.001	2.086	0.461	<0.001	0.526	0.376	0.162	0.895	0.143	<0.001
Experiencing a decline in personal health	2.345	0.196	<0.001	2.110	0.226	<0.001	0.944	0.187	<0.001	0.492	0.072	<0.001
Financial difficulty	0.734	0.203	<0.001	0.947	0.230	<0.001	-0.036	0.187	0.846	0.123	0.072	0.089
Conflict with parents	1.312	0.247	<0.001	1.575	0.280	<0.001	0.601	0.228	0.009	0.417	0.088	<0.001
Death of a close family member	0.510	0.312	0.102	0.957	0.353	0.007	0.214	0.286	0.455	0.276	0.110	0.012
Difficulty participating in social activities	1.056	0.200	<0.001	1.287	0.226	<0.001	0.800	0.184	<0.001	0.205	0.071	0.004
Feeling uncomfortable with living situation	1.447	0.215	0.000	1.750	0.243	<0.001	0.713	0.200	<0.001	0.549	0.076	<0.001

## Discussion

The present study showed the high prevalence of depression in medical students in Northern Vietnam and the appreciable effect of medical education, relationships, life events and personal health on depression. To the best of our knowledge, this is one of very few studies on depression in Vietnamese medical students and the first study that has investigated the association between depressive symptoms and related factors, particularly for each of the four domains of the CES-D scale.

Recently, an increasing number of studies have evaluated depression and the mental health of medical students. Overall, these studies have consistently suggested that levels of depression among medical students are higher than among the general population and that female medical students have higher levels of depression than male students[9, 24]. Inversely, other studies have suggested that among medical students, females were not more vulnerable to depression than males[25, 26]. In the current study, 39% of the medical students appeared to be experiencing symptoms of depression, which was higher than the prevalence found in another study carried out in the general young population of Vietnam[27]. The current study also found a higher prevalence of depression than a previous study among Vietnamese medical students[28]. A meta-analysis study by Rotenstein LS using data from 167 cross-sectional studies and 16 longitudinal studies from 43 countries estimated the prevalence of depression or depressive symptoms among medical students as 27.2%; the prevalence among Asian medical students was 29.1%[29]. Those findings were lower than ours. Other meta-analysis study by Puthran that extracted data from 77 studies also demonstrated a global prevalence of depression among medical students of 28.0%; the prevalence in Asian countries was 30.1% [30]. The difference may be due to using different tools to evaluate depressive symptoms in each study. Additionally, Rotenstein reported that low- and middle-income regions (Africa, the Middle East) had a higher prevalence of depression (46.3%, 35.2%), which is in line with the trend towards a higher prevalence of depression in Middle Eastern students reported in Puthran’s study. Along with our findings, those results suggest that income might be an important depression-related factor. Furthermore, this study did not find a trend toward a higher prevalence of depressive symptoms in female students; on the contrary, we found that the prevalence was higher in males. One possible explanation for this inconsistency is the difference in the instruments used to assess depression. However, our results were in line with the findings of other studies that used the CES-D questionnaire; those studies showed that more than 30% of American students [31] and 37% of Korean students[32] experience depression. These reported values are higher than those of other studies, including studies at Albert Einstein College of Medicine[33] and studies conducted among female students in Israel[34] and in Hawaii[35]. However, they are lower than the prevalence of depressive symptoms reported for Chinese students[36] and North American[37] students.

A number of studies have examined the contribution of depression-related factors to medical students’ mental health. In addition to the rigors of training, medical students can face major personal life events, such as illnesses and death of family members. Such personal factors are known to contribute to depression, anxiety and substance use in the general population[38–40]. A study of Turkish medical students showed that depression is associated with dissatisfaction with social activities, worry about having made the wrong career choice, accommodation problems, education failures and worries about the future[41]. The findings related to these risk factors in our study support similar associations in previous studies. Of these factors, those that affected educational progress, such as being absent from many classes and conflicts with teachers, as well as daily life-related factors, such as conflicts with parents, difficulties participating in social activities, discomfort with one’s living situation and declines in personal health, were correlated with depression in all four domains. The development of close relationships between students and lecturers and between children and parents is an important solution for reducing symptoms of depression in students. The relatively high correlation between the somatic complaints and the depressive affect domains found in the current study suggests that these two domains have similar relationships with risk factors and stronger codependent relationships than the other structure factors. However, the level of impact that each risk factor had on individual domains was different. Similar to somatic complaints and depressive affect, interpersonal problems had a significant relationship with most of the risk factors; the exceptions were lower grades than expected, achieving excellence in studies and financial problems. Students who said that they would choose a medical career again had a higher risk of depression; however, this factor was only correlated with interpersonal problems of feeling unfriendliness or dislike from others. Being a regular student in medical school in Vietnam, as in other countries, is very difficult. However, before high school students take medical school admissions examinations, they do not receive any information about the course of training in medical school or the differences among specializations. Therefore, many students misunderstand their majors and lose their sense of direction when studying. Additionally, the medical education and health systems in Vietnam still have limitations compared with those in other countries; consequently, medical schools are changing their curriculums to more closely resemble those of international medical schools. These changes strongly affect students’ willingness to choose the same career again given the chance. Thus, the development of stable education system that is suitable to Vietnam’s situation is an urgent requirement. Conflicts with teachers had the second strongest impact on depression. This factor had the strongest effect on positive affect, the second strongest effect on interpersonal problems and a lower effect on the two remaining domains. Having a university policy offense showed a high correlation with depression, but it was not significantly associated with positive affect. Financial problems have been identified as a possible risk factor for depression in several studies[38, 42]. In the present study, financial problems were one of two factors that did not affect depression. However, when each domain was analyzed, significant correlations between financial problems and depression were found in the somatic complaints and depressive affect domains. These results support the conceptual distinctions among the four domains. Because this is the first study to evaluate the specific associations between each of the four domains of the CES-D scale and related risk factors for depression in medical students, there is no comparable data available in the literature. Nevertheless, our analyses may imply that depression-related factors do not influence the four domains of the CES-D scale in exactly the same way. Therefore, to reduce the prevalence of depression among medical students, particularly students at HPUMP, Vietnam, we should consider each factor carefully and apply suitable solutions, such as developing a student union or clubs or increasing social activities for students.

It is essential to consider the limitations of the data and the analysis in this study. This was a cross-sectional study that used an epidemiological scale as a self-assessment measure without any confirmation from clinical physicians. Moreover, in the present study, we only identified depressive symptoms using a cut-off score of 16 and did not evaluate the level of depression; therefore, we could not show the association between risk factors and the severity of depression more clearly. Because the study was performed at one point in time and used convenience sampling with a lack of baseline information on mental status, our results cannot be used to draw conclusions about causal relationships and how distress in medical students changes throughout the course of training. Future longitudinal studies should focus on other ways to obtain more precise results.

## Conclusions

This was the first study to investigate the association between the four domains of the CES-D and risk factors for depression in medical students. The study found that various risk factors have different impacts on each domain of depression. The present study clarified the influence of risk factors on depression in hopes of make better recommendations to reduce the prevalence of depression in medical students.

## Supporting information

S1 Table Associations between participants’ characteristics and depressive symptoms. (DOCX)
