# Supplementary material for: The four-domain structure model of a depression scale for medical students: A cross-sectional study in Haiphong, Vietnam
Source: PLoS One. 2018 Mar 22;13(3):e0194550. doi: 10.1371/journal.pone.0194550 (PMC5864022; doi:10.1371/journal.pone.0194550)
Supplement: S1 Table — (DOCX) [file pone.0194550.s001.docx]

# **Supporting information**

**S1 Table. Associations between participants’ characteristics and depressive symptoms**

| Characteristics | | **Univariate** | | | | | **Multivariate^*^** | | | | |
| --- | --- | --- | --- | --- | --- | --- | --- | --- | --- | --- | --- |
|  |  | B | OR | 95% C.I.for OR | | p | B | OR | 95% C.I.for OR | | p |
|  |  |  |  | Lower | Upper |  |  |  | Lower | Upper |  |
| Gender^a^ | Female | Reference | | | | | | | | | |
|  | Male | -0.303 | 0.739 | 0.579 | 0.943 | 0.015 | -0.347 | 0.707 | 0.550 | 0.908 | 0.007 |
| Age (years) | | -0.013 | 0.987 | 0.927 | 1.051 | 0.678 | 0.027 | 1.027 | 0.920 | 1.147 | 0.636 |
| Ethnicity^a^ | Kinh | Reference | | | | | | | | | |
|  | Other | 0.278 | 1.320 | 0.826 | 2.109 | 0.246 | 0.339 | 1.404 | 0.860 | 2.292 | 0.175 |
| Religion^a^ | None | Reference | | | | | | | | | |
|  | Religious | 0.309 | 1.363 | 0.919 | 2.021 | 0.124 | 0.241 | 1.273 | 0.851 | 1.903 | 0.240 |
| Grade level^a^ | Pre-clinical | Reference | | | | | | | | | |
|  | Clinical | -0.105 | 0.901 | 0.721 | 1.125 | 0.356 | -0.092 | .912 | 0.727 | 1.144 | 0.427 |
| Area^a^ | Urban | Reference | | | | | | | | | |
|  | Suburb | -0.048 | 0.953 | 0.755 | 1.205 | 0.689 | 0.009 | 1.009 | 0.783 | 1.302 | 0.943 |
| Living condition | With family | Reference | | | | | | | | | |
|  | Dorm | -0.150 | 0.861 | 0.545 | 1.359 | 0.520 | -0.265 | 0.767 | 0.476 | 1.236 | 0.276 |
|  | Rental house | -0.279 | 0.757 | 0.561 | 1.022 | 0.069 | -0.348 | 0.706 | 0.512 | 0.973 | 0.034 |
| Marriage status^a^ | Single | Reference | | | | | | | | | |
|  | Other | 0.307 | 1.359 | 0.779 | 2.372 | 0.280 | 0.226 | 1.254 | 0.709 | 2.218 | 0.437 |
| Specialization^a^ | General doctor | Reference | | | | | | | | | |
|  | Other | -0.137 | 0.872 | 0.694 | 1.096 | 0.240 | -0.183 | 0.833 | 0.657 | 1.056 | 0.131 |
| Fathers’ educational background ^a^ | >High school | Reference | | | | | | | | | |
|  | ≤Primary school | 0.178 | 1.195 | 0.726 | 1.966 | 0.484 | -0.104 | 0.901 | 0.502 | 1.617 | 0.727 |
|  | Secondary school | 0.306 | 1.358 | 1.018 | 1.812 | 0.038 | 0.270 | 1.310 | 0.918 | 1.870 | 0.136 |
|  | High school | 0.057 | 1.059 | 0.791 | 1.416 | 0.701 | 0.081 | 1.084 | 0.777 | 1.512 | 0.635 |
| Mothers’ educational background ^a^ | ≤Primary school | Reference | | | | | | | | | |
|  | Secondary school | -0.486 | 0.615 | 0.381 | 0.995 | 0.047 | -0.615 | 0.541 | 0.316 | 0.925 | 0.025 |
|  | High school | -0.639 | 0.528 | 0.325 | 0.857 | 0.010 | -0.664 | 0.515 | 0.298 | 0.888 | 0.017 |
|  | >High school | -0.674 | 0.510 | 0.309 | 0.841 | 0.008 | -0.685 | 0.504 | 0.280 | 0.905 | 0.022 |
| Parents’ marital status^a^ | Living together | Reference | | | | | | | | | |
|  | Other | -0.067 | 0.935 | 0.609 | 1.436 | .759 | -0.105 | 0.900 | 0.580 | 1.397 | 0.640 |
|  | ^*^Adjusted for all characteristics variable which given the p for constant <0.05 in univariate logistic regression analysis  ^a^p for constant <0.05 in univariate analysis | | | | | | | | | | |
